# Analysis of the *Saccharomyces cerevisiae *proteome with PeptideAtlas

**DOI:** 10.1186/gb-2006-7-11-r106

**Published:** 2006-11-13

**Authors:** Nichole L King, Eric W Deutsch, Jeffrey A Ranish, Alexey I Nesvizhskii, James S Eddes, Parag Mallick, Jimmy Eng, Frank Desiere, Mark Flory, Daniel B Martin, Bong Kim, Hookeun Lee, Brian Raught, Ruedi Aebersold

**Affiliations:** 1 Institute for Systems Biology, N 34th Street, Seattle, WA 98103, USA; 2 Department of Pathology, University of Michigan, Catherine Road, Ann Arbor, MI 48109, USA; 3 Louis Warschaw Prostate Cancer Center, Cedars-Sinai Medical Center, W. Third St, Los Angeles, CA 90048, USA; 4 PHSD, Fred Hutchinson Cancer Research Center, Seattle, WA 98109, USA; 5 Nestlé Research Center, 1000 Lausanne 26, Switzerland; 6 Department of Molecular Biology and Biochemistry, Wesleyan University, Middletown, CT 06459, USA; 7 Divisions of Human Biology and Clinical Research, Fred Hutchinson Cancer Research Center, Seattle, WA 98109-1024, USA; 8 IMSB, ETH Zurich and Faculty of Science, University of Zurich, Zurich, Switzerland; 9 University Health Network, Ontario Cancer Institute and McLaughlin Centre for Molecular Medicine, College Street, Toronto, ON M5G 1L7, Canada

## Abstract

We present the *Saccharomyces cerevisiae *PeptideAtlas composed from 47 diverse experiments and 4.9 million tandem mass spectra. The observed peptides align to 61% of *Saccharomyces *Genome Database (SGD) open reading frames (ORFs), 49% of the uncharacterized SGD ORFs, 54% of *S. cerevisiae *ORFs with a Gene Ontology annotation of 'molecular function unknown', and 76% of ORFs with Gene names. We highlight the use of this resource for data mining, construction of high quality lists for targeted proteomics, validation of proteins, and software development.

## Abstract 2

The *S. cerevisiae *PeptideAtlas, composed from 47 diverse experiments and nearly 5 million tandem mass spectra, is described.

## Background

The field of genomics is slowly reaching maturity. The genomes of many organisms have now been sequenced and the effort to annotate these genomes is now well underway. Transcriptomes are routinely investigated, as mRNA expression can be measured with highly sensitive microarrays and other methods. In contrast, the measurement and annotation of proteomes remains challenging. Proteome analysis is primarily based on mass spectrometry (MS) and is not as mature as gene expression analysis. However, proteomic measurements are preferable in some situations because, while mRNA expression studies indicate the potential for protein expression, they do not directly measure proteome characteristics. For example, mRNA expression levels do not always correlate well with protein expression levels due to variations in translation efficiencies [1] and targeted degradation of proteins in the cell [2,3]. Additionally, proteins are subjected to numerous post-transcriptional modifications that alter the chemical composition of the protein. Proteins also interact with other proteins in a highly dynamic way.

Proteomics by MS has emerged as an effective tool for probing those properties of expressed genes that are not directly apparent from the mRNA sequence or transcript abundance, including the subcellular location of a protein of interest [4-6], the identification of post-translational modifications [7,8], the characterization of interacting proteins or ligands [9], and the measurement of changes in these protein properties throughout the cell cycle or in response to a given stimuli or stress [10-12]. Coupled with various types of isotopic labeling reagents, MS can also be used to directly determine relative and absolute protein abundances [13]. Abundance measurements in proteomics are difficult, however, compared to mRNA studies as there are no amplification strategies such as PCR to increase the concentration of low abundance analytes.

In the present study, we attempt to characterize the *Saccharomyces cerevisiae *proteome using MS based proteomics. *S. cerevisiae *is a widely used and important model organism with a relatively large, but structurally simple, genome for which a high quality and well annotated sequence is available. It exhibits many of the same pathways and cellular functions as higher Eukaryotes. The largest published *S. cerevisiae *protein expression study used epitope tagging to detect 73% of the annotated *Saccharomyces *Genome Database (SGD) open reading frames (ORFs), which is 83% of SGD ORFs with Gene names [14,15]. Another recent study identified 72% of the predicted yeast proteome [16]. In this paper we combine the data from 47 different MS experiments that collectively generated 4.9 million spectra, into a single structure, the *Saccharomyces cerevisiae *PeptideAtlas.

The PeptideAtlas Project provides software tools and an infrastructure for the integration, visualization and analysis of multiple MS datasets [17-19]. This resource can be used to design future, more efficient experiments, to assist in the exploration of the proteome, and to support the development of proteomics software by making the data publicly accessible. We additionally demonstrate how this resource can be used in the construction of high quality lists of observable peptides for synthesis as reference molecules for targeted proteomics. This novel resource improves as more researchers contribute datasets.

## Results and discussion

### PeptideAtlas construction

The *S. cerevisiae *PeptideAtlas is composed of 47 datasets (Table 1) from many different sources that were generated by using a variety of protocols and separation techniques. All samples in this atlas were proteolytically digested with trypsin, and many were treated with one of the isotope-coded affinity tagging (ICAT) reagents [10] or iodoacetamide. All samples were acquired using LC-ESI instruments (liquid chromatography separation, and electrospray ionization coupled with MS) - no matrix-assisted laser desorption ionization time-of-flight (MALDI-TOF) instrument datasets were available for inclusion. The PeptideAtlas can, however, accept and be expanded with data from any type of instrument using the mzXML data format (see below). A variety of protein or peptide separation techniques were employed in these experiments, including SDS-PAGE, free-flow electrophoresis and strong cation exchange chromatography, to generate fractions that were then subjected to reversed-phase HPLC separation prior to mass spectrometric analysis.

**Figure 1:**
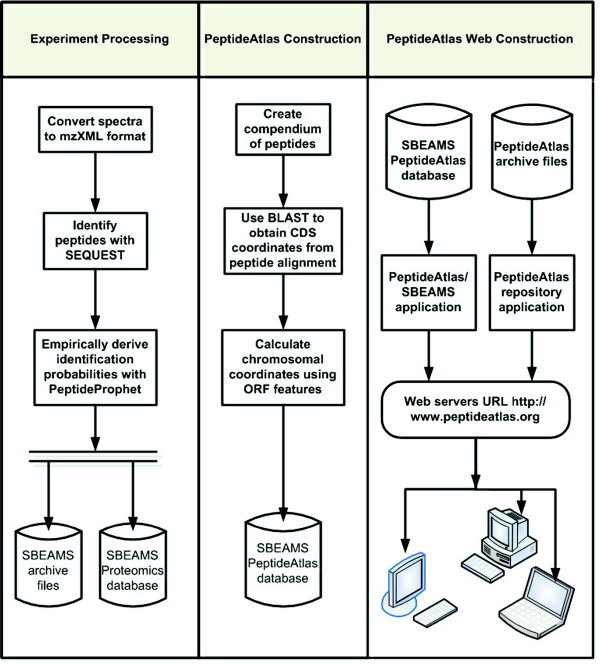
PeptideAtlas processing, creation, and interfaces. The first column outlines experiment level processing with SEQUEST [21] and PeptideProphet [22], the second column shows major stages in the construction of a PeptideAtlas [18] using BLAST [24] to obtain coding sequence (CDS) coordinates, and the third column shows the data, business logic, and presentation tiers for a PeptideAtlas.

Processing of the acquired spectra and construction of a PeptideAtlas are briefly summarized in Figure 1 and described in more detail in previous publications [17-19]. For each submitted yeast dataset, all spectra were first converted to the mzXML format [20] irrespective of the original file format and then searched using SEQUEST [21] against a non-redundant *S. cerevisiae *reference protein database (the union of the SGD, Ensembl, NCI, and GenBank databases as detailed in Additional data file 1, plus keratin and trypsin). Redundant ORF sequences were coalesced to single entries with combined description fields. The union of the five protein sequence files yielded 13,748 distinct ORF sequences. Many ORF sequences differed by only a few amino acid residues, but all differences were retained in order to maximize the number of sequence assignments.

For each experiment the primary database search results were assigned statistical probabilities using the PeptideProphet program [22] implemented in the Trans-Proteomic-Pipeline [23]. Table 2 lists the number of spectra per experiment in total and with a PeptideProphet assignment P ≥ 0.9, and the number of distinct peptide identifications cumulatively added by each experiment. The experiments are sorted approximately in the order submitted; the latter experiments will naturally make a smaller contribution to the total list of distinct peptides as many of the peptides identified in the latter experiments were also identified in earlier experiments.

Search results and spectra were stored in the Systems Biology Experiment Analysis Management System (SBEAMS), and all files were retained in an archive area.

To create the *S. cerevisiae *PeptideAtlas, all peptide information in contributed datasets was filtered to retain only those identifications with a PeptideProphet probability above 0.9. The remaining peptide sequences were then aligned with the SGD reference protein database using the NCBI program BLASTP [24] with arguments to achieve the highest scores for identities of 100% without gaps. Chromosomal coordinates were then calculated using the BLAST-provided coding sequence (CDS) coordinates of the peptide combined with the chromosomal coordinates of the protein features (where features are the contents of the SGD_features table that contain elements present in the GFF3 guidelines [25]). Peptide information along with the chromosomal coordinates were loaded into the PeptideAtlas database.

Summary statistics of the current yeast PeptideAtlas build are presented in Table 3. The build was constructed with a lower limit threshold of peptide assignments with P ≥ 0.9, but the build may be searched with higher thresholds and those summary statistics are also reported in Table 3. The number of distinct peptide sequences identified in these spectra (with P > 0.9) is 36,133. The number of SGD proteins with which these peptides display perfect alignment is 4,063, which is 61% of all ORFs in the SGD protein database. If we apply a stricter criteria of removing identifications in which a peptide was only observed once in the entire *S. cerevisiae *PeptideAtlas, we then find that 43% of all SGD ORFs have been seen (with P > 0.9). If we also apply another criterion, that we count only the peptide to protein mappings that are not degenerate and that have P ≥ 0.9, we observe 59% of SGD ORFs. The same criteria applied to peptides that have been seen more than once results in an observation of 41% of SGD ORFs. The number of peptides with perfect alignment to protein sequences in files other than SGD is 110 (Additional data file 1). Some of these identifications correspond to records that NCBI has discontinued or are identifications to appended contaminant sequences such as keratin or trypsin identified in the search, but are not present in the target genome (*S. cerevisiae *in this case).

Expected errors are calculated with equation 14 of the PeptideProphet paper using the summation of (1 - P_i_) divided by N_i_. This is applied to four cases, summarized as rows in Table 4, and three PeptideProphet probability limits, shown as columns in Table 4. The cases are: one, the assigned probability P of each MS/MS is used for all P_i _≥ P_limit_; two, the assigned probability P of each MS/MS is used for all P_i _≥ P_limit _where the associated peptide has been seen in the *S. cerevisiae *PeptideAtlas more than once; three, the best probability for each unique peptide sequence is used for all P ≥ P_limit_; and four, the best probability for each unique peptide sequence is used for all P ≥ P_limit _when the peptide has been seen in the *S. cerevisiae *PeptideAtlas more than once. Note that cases three and four make an assumption that the peptide identifications can be represented by the best identification probability for that peptide. There are many methods to score groups of peptides, and we adopt the simplest in this paper for cases three and four as they represent the data as we have used it. For more detailed discussions on group scoring, please see [26,27]. Table 4 shows that the expected error rate for the *S. cerevisiae *PeptideAtlas as a whole is 9%. As an aside, one can construct subsets of the build with smaller error rates by using a higher PeptideProphet probability threshold (see changes along a row in Table 4) or by reducing the number of peptides one is counting (see changes along a column in Table 4) by only using those peptides that have been observed more than once, and further removing information from multiple instances of those peptides.

In summary, the *S. cerevisiae *PeptideAtlas expected error rate is 9%, but the user is able to construct subset exports of the build with reduced error rates if desired.

### To what extent do the peptides in the *S. cerevisiae *PeptideAtlas represent the *S. cerevisiae *proteome?

The coverage of the *S. cerevisiae *proteome by the PeptideAtlas is high, but not complete. Using only peptide identifications possessing a PeptideProphet score of P > 0.9, we have mapped to 61% of all SGD ORFs with at least one peptide hit. In Table 5 we present the observed ORFs categorized by feature type annotations. Of the 'uncharacterized' ORFs, 49% are represented in the *S. cerevisiae *PeptideAtlas (Additional data file 2). Uncharacterized ORFs are defined as putative gene products with homologs in another species that have, however, not been experimentally observed in *S. cerevisiae*. Of the 'verified' ORFs, 74% are represented in the PeptideAtlas. Verified ORFs are those that have been experimentally confirmed to exist in *S. cerevisiae*. A small percentage of ORFs are annotated as 'dubious'; only very few of these ORFs were found in the PeptideAtlas. Dubious ORFs are putative gene products that do not have homologs in other *Saccharomyces *species, and for which there is no experimental evidence of existence in *S. cerevisiae*. (Additional data file 3). Of the ORFs annotated as pseudogenes, 19% are represented in the PeptideAtlas. An SGD pseudogene has a functional homolog in another organism, and is predicted to no longer function because mutations prevent its transcription or translation. The pseudogene classification is based upon observations of ORFs from the S288C strain. Of the ORFs annotated as transposable elements, 20% are present in the PeptideAtlas (Additional data file 4). Note that the coverage of the ORFs in these categories decreases if we remove those peptides that have only been observed once in the entire atlas. With the single hit peptides removed, we see 56% of SGD verified ORFs in PeptideAtlas, 29% of the uncharacterized ORFs, none of the ORFs from verified/silenced_genes, 2% of the dubious ORFs, 5% of ORFs from pseudogenes, and 15% of ORFs from genes categorized as transposable element genes. The assignments to dubious ORFs should be viewed skeptically as the numbers of observations are extremely small and within error of the atlas.

**Figure 2:**
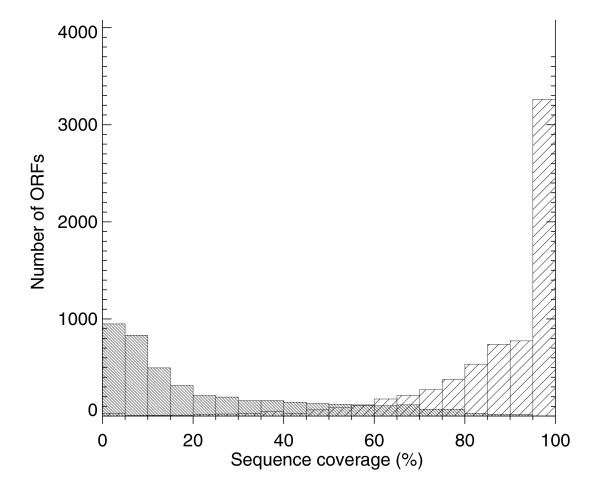
Histrograms of protein sequence coverage. A histogram of the sequence coverage of the *S. cerevisiae *ORFs by PeptideAtlas (darker filled bars) and an *in silico *tryptic digestion of the reference protein database with a mass range of 500 to 4,000 Da (lighter diagonal pattern filled bars) is shown. Of the PeptideAtlas ORFs, 61% have coverage below 20%, while 39% have a coverage above 20%.

A histogram of ORF sequence coverage is shown in Figure 2; the PeptideAtlas distribution is represented by the shaded distribution on the left, while an *in silico *digested SGD reference ORF distribution filtered to retain peptides with average molecular weights between 500 and 4,000 Da is seen on the right. About 40% of the ORFs in PeptideAtlas have sequence coverage greater than 20%. Importantly, the entire yeast proteome is not expected to be observable by current tandem MS techniques. This is not an impediment to protein identification, as the entire set of measurable peptides for a given protein is not necessary for an unambiguous identification of the protein. Some of the reasons that perfect sequence coverage is not possible are inherent in the instruments (discussed further in the next section) and in the search techniques. For example, we may miss identifications of sequences from post-translationally modified proteins in the search strategy applied.

### Biases in the *S. cerevisiae *PeptideAtlas

Since all the data in the PeptideAtlas were acquired using LC-ESI-MS/MS, we examined peptide hydrophobicity, mass and charge distributions to characterize any inherent biases in the peptide dataset.

**Figure 3:**
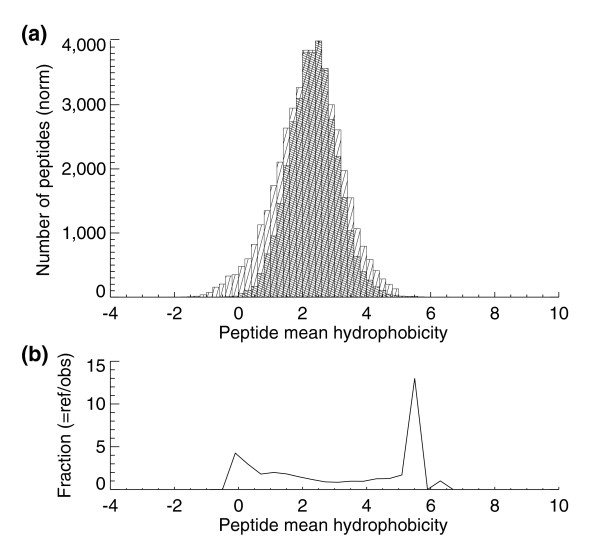
Hydrophobicity histrogams. **(a) **Mean hydrophobicity histogram for peptides in the *S. cerevisiae *PeptideAtlas (darker hashed bars) and an *in silico *tryptic digest of the SGD reference protein database allowing one missed cleavage (lighter hashed bars). **(b) **The reference peptides' hydrophobicities divided by the observed peptides' hydrophobicities. The lowest hydrophobicity peptides are generally washed off the column in the reverse phase stage of the HPLC process and hence not measured.

Using the Guo [28] and Krokhin *et al*. parameters [29] we created hydrophobicity histograms for the *S. cerevisiae *PeptideAtlas peptides (darker hatched bars), overlaid on an *in silico *digest of the entire SGD database (lighter hatched bars) allowing one missed tryptic cleavage (Figure 3). While peptides of moderate hydrophobicity were efficiently observed, the *S. cerevisiae *PeptideAtlas is clearly lacking in the most hydrophilic peptides - presumably because these peptides do not efficiently bind to standard HPLC columns and proceed to waste instead of entering the mass spectrometer. Other types of upstream separation techniques or modification of HPLC solvent conditions will most likely be required to improve the detection of these hydrophilic peptides. Similarly, the most hydrophobic peptides are also not as efficiently observed as peptides with more moderate hydrophobicity scores, presumably because these peptides do not elute efficiently under standard LC gradient conditions.

**Figure 4:**
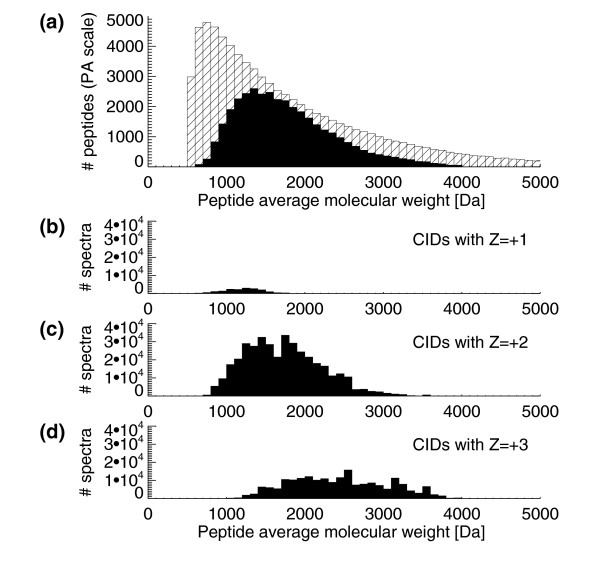
Mass histograms. **(a) **Average molecular weight of unique peptide sequences in PeptideAtlas (solid filled bars) and the *in silico *tryptically digested SGD protein database (hashed bars) allowing one missed cleavage. **(b-d) **Mass histograms of spectra, separated by charge. The large number of peptides with masses less than 1,000 Da are difficult to identify in database searches, and hence are not present in the PeptideAtlas. CID, collision-induced dissociation.

A bias is also present in the distribution of peptide masses. Figure 4 shows histograms of *S. cerevisiae *PeptideAtlas average molecular weights (solid bars) overlaid on an *in silico *digest of the entire SGD database (hatched bars). The acquisition settings for MS/MS instruments are typically in the range of 400 to 2,000 m/z which, accounting for charge states, limits the peptide mass detection range to 400 to 6,000 Da. The database searches, however, have a range of roughly 600 to 4,200 Da. The *in silico *digest reference distribution suggests that there would be a peak at a mass of approximately 700 amu, but the observed peak is near 1,500 amu. In the PeptideAtlas, we appear to be missing many of the peptides with masses less than 1,400 Da, largely because these smaller peptides are more difficult to identify using standard database search tools. Importantly, however, smaller peptides are often not as useful in protein identification as the longer peptides, since the short amino acid sequences are less likely to be unique to a single protein. Additionally, larger peptides tend to have more than one chance of being observed, as charge states of +2 or +3 can put the peptides within range of the acquisition settings of the instrument. Figure 4 shows that there are a larger number of peptides in charge states of +2 and +3, with only a small percentage of peptide identifications derived from a +1 charge state. This is expected given that the datasets in this version of the PeptideAtlas are from ESI instruments. We do not currently search the spectra for ions with charge states larger than +3 and it could be expected that many of the missed larger peptides might generate higher charge state ions. The addition of MALDI-TOF datasets to the atlas will populate the database with identifications from ions in the +1 charge state. Krogan *et al*. [16] find high protein discovery rates using MALDI, so this approach is promising.

In summary, a sizable fraction of the yeast proteome has been identified using LC-ESI MS/MS. The smallest peptides are not well represented in the PeptideAtlas. Additionally, the most hydrophilic peptides and hydrophobic peptides are under represented. It will be interesting to determine how these distributions change as more diverse types of data are added to the atlas.

### The relationship between number of spectra and number of identified peptides

**Figure 5:**
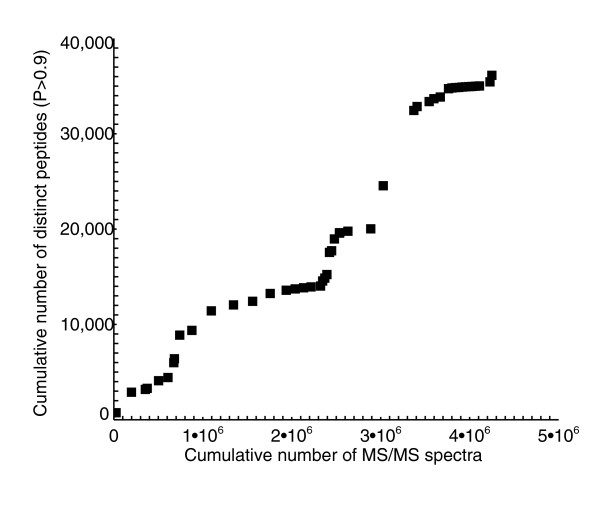
Cumulative number of MS/MS spectra versus the number of unique peptide identifications with P > 0.9. The slope is nearly horizontal in regions of the curve where similar experiments were performed. The curve is expected to show saturation when additional spectra provide no new peptides above P > 0.9. The number of distinct peptides from an *in silico *trypic digestion of the SGD protein database, allowing one missed cleavage, and counting those peptides whose masses are in the range 500 to 4,000 Da, is 436,445.

As the PeptideAtlas is continually populated with new datasets, we expect that, at some stage, the addition of new spectra will produce few new peptide identifications. We are thus tracking the number of unique peptides contributed by each additional experiment. From a total of 4.9 million MS/MS spectra, we have 36,133 distinct peptides with PeptideProphet scores >0.9 (Table 3). As an aside, the number of distinct peptides from an *in silico *tryptic digestion of the SGD protein database, allowing one missed cleavage, and counting those peptides whose masses are in the range 500 to 4,000 Da, is 436,445, so we currently observe roughly 10% of what we might expect if all peptides had equal possibility of being observed. The current rate of inclusion of unique peptide identifications with P > 0.9 is shown in Figure 5. In general, there is one peptide added for every 125 spectra. Flattened areas of the curve are due to overlapping identification of peptides from similar experiments (and instrument), rather than the expected final trend of saturation of the proteome sequences. Remarkable increases in distinct peptide yields are seen from the Gygi *et al*. [10] yeast dataset and the Ho *et al*. [30] gel_msms dataset. In summary, we have identified roughly ten percent of the peptides predicted from an *in silico *digested protein database, and have not yet reached saturation of novel additions. Novel additions are expected with the inclusion of results from new experiment designs and instrument platforms.

### What new information does the PeptideAtlas contribute about the *S. cerevisiae *proteome and genome?

**Figure 6:**
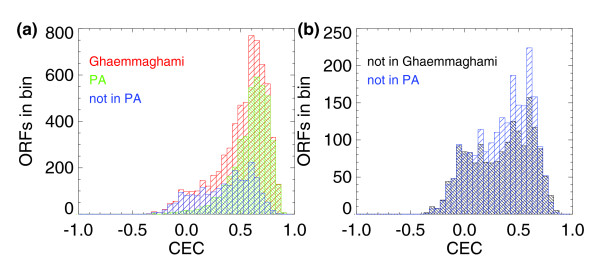
Comparison of codon enrichment correlation (CEC) distributions. **(a) **Histogram of CEC for all ORFs listed in Ghaemmaghami *et al*. [14], for all ORFs seen in PeptideAtlas (PA), and for all ORFs not seen in PeptideAtlas; and **(b) **histogram of CEC for ORFs not seen in the Ghaemmaghami *et al*. expression sets and for all ORFs not seen in PeptideAtlas. Observed proteins in PeptideAtlas and in Ghaemmaghami *et al*. show a positive skew in CEC showing that they deviate significantly from that expected from a random sequence of codons, while the unobserved proteins show more uniformly distributed CEC values.

Having characterized some observational biases in peptide content of the PeptideAtlas, we now briefly examine characteristics of the identifications in relation to predictions based on codon bias and gene ontology categories. Figure 6 illustrates the codon enrichment correlation (CEC) for all ORFs in the *S. cerevisiae *PeptideAtlas and all ORFs in Ghaemmaghami *et al*. [14]. CEC is a parameter constructed by Ghaemmaghami *et al*. [14] to represent a measurement of the deviation of observed protein codon-usage from codon-usage in a randomly generated ORF. These distributions are both skewed toward high positive values, typically greater than 0.25, signifying that their sequences deviate significantly from that of ORFs derived from random codons; these are thus likely to be true ORFs and not spurious predictions. Figure 6b is a histogram of CEC for ORFs not observed in the Ghaemmaghami *et al*. expression sets, and for all ORFs not present in PeptideAtlas. Their distributions are evenly distributed around the origin, suggesting that both datasets are missing *bona fide *ORFs as well as ORFs that are not likely to code for proteins.

**Figure 7:**
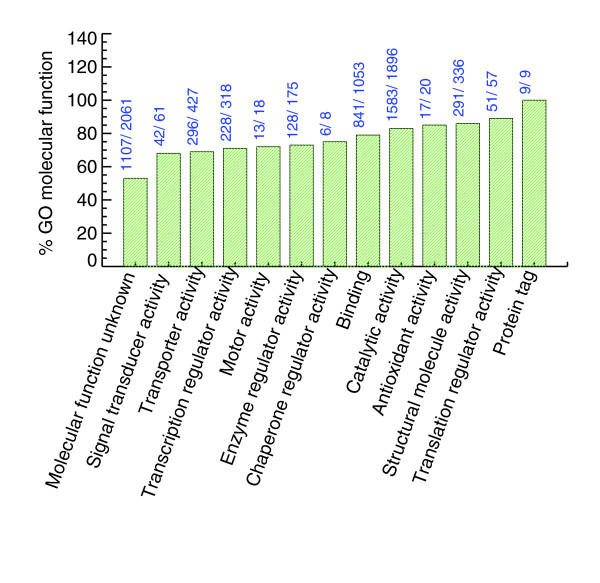
The number of genes identified in GO Molecular Function terms (more specifically, the first level children of molecular function). The bars are annotated with the number of SGD genes annotated in that term and the number of SGD genes seen in PeptideAtlas for that term. Many annotated as unknown are present in the PeptideAtlas.

**Figure 8:**
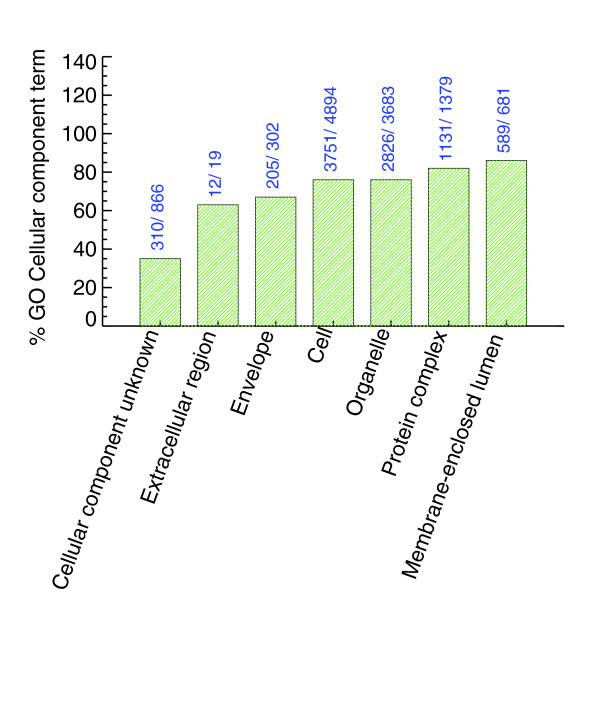
The number of genes identified in GO Cellular Component terms (more specifically, the first level children). The bars are annotated with the number of SGD genes and the number of SGD genes in PeptideAtlas for that term. Many annotated as unknown are present in the PeptideAtlas.

**Figure 9:**
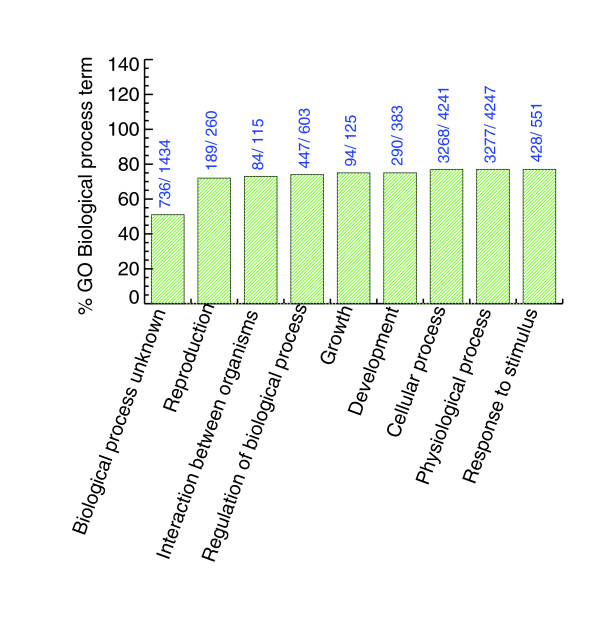
The number of genes identified in GO Biological Process terms (more specifically, the first level children). The bars are annotated with the number of SGD genes and the number of SGD genes in PeptideAtlas for that term.

The proteins identified in the yeast PeptideAtlas are generally evenly distributed with respect to Gene Ontology (GO) molecular function categories (Figure 7). Interestingly, we observe 52% of yeast ORFs annotated as 'molecular function unknown'. If we filter out peptides that have been observed only once in the PeptideAtlas, the percent of 'molecular function unknown' genes we see is 32% (approximately 659 genes). The same trend is seen in the GO 'cellular components' and the GO 'biological processes' categories. Figures 8 and 9 show that a significant fraction of ORFs in PeptideAtlas are present in the catgories 'cellular component unknown' and 'biological process unknown'.

The *S. cerevisiae *PeptideAtlas, by verifying that these unannotated ORFs produce identifiable proteins, could stimulate interest in determining their function.

### PeptideAtlas user interface

Summary statistics and query interfaces for the PeptideAtlas are available at [31]. Perl cgi-bin programs and Java servlets and JSP pages enable public queries of the database and public archive area flat files. From the PeptideAtlas front page [31], atlas data may be accessed by links on the navigation bar. The 'Data Repository' link leads to a form where the user can retrieve all publicly available datasets and search results. The 'Search Database' link leads to several pages where the user can either search the atlas for keywords, find the information summary for a given peptide in the atlas, or produce a tailored list of peptides from the atlas by specifying a variety of constraints.

The *S. cerevisiae *PeptideAtlas user interface thus allows for an extremely large number of diverse tandem MS datasets to be searched, processed, and combined in a user-specifiable fashion.

### Uses of the *S. cerevisiae *PeptideAtlas

The *S. cerevisiae *PeptideAtlas represents an extremely useful data mining tool. Here, we present two examples of how the database may be used. The first example demonstrates the creation of a list for construction of synthetic peptides, and the second example demonstrates validation of predicted SGD introns.

Using quantitative MS techniques, synthetic peptides may be utilized to determine subunit stoichiometry within a given multi-protein complex [32] or to determine the absolute quantity of a protein in a sample [33,34]. To design such peptides, one could use the *S. cerevisiae *PeptideAtlas to: identify those peptides of member proteins that have been observed most often in the mass spectrometer; identify those that are specific for a single protein; and determine which of these peptides contain amino acids necessary for a given type of labeling reagent and suitable for peptide synthesis. For example, if the stoichiometry of the general transcription factor TFIIF complex was to be determined, the user would go to the PeptideAtlas URL [31], enter %tfIIf% into the search box and select 'GO'. The results are links to PeptideAtlas pages corresponding to the three SGD ORFs that are part of the TFIIF transcription factor complex in this organism [35,36]. One may either follow the links to the individual pages for the three ORFs, or open a new 'Browse Peptides' page using the tab located near the top of the page to query the atlas in more detail. In the new 'Browse Peptides' page, the user can enter the three SGD ORFs into the 'Protein Name Constraint' text box (separated by semi-colons: YPL129W; YGR186W; YGR005C). Further useful constraints to enter are '>0.9' for 'Best Probability Constraint', '>1' for 'Number of Observations Constraint', and ' = 1' for 'Number of Proteins Mapped Constraint'. Following selection of 'Query' near the bottom of the page, results are returned below the form. To tailor the list to ICAT experiments, one could also enter %C% in the 'Peptide Sequence Constraint' text box above and select 'Query' again. The returned list consists of five cysteine-containing peptides whose sequences have been observed more than once with high confidence, and which are present in only one protein. The resulting list may be exported to the user's desktop in Excel, xml, tsv, or csv file formats.

As an example of a bioinformatics use of the *S. cerevisiae *PeptideAtlas, one could search for all experimentally observed splice forms predicted in the SGD database that are present in the *S. cerevisiae *PeptideAtlas. In this case, the user would select 'Search Database' from the navigation bar on the left, select the latest *Saccharomyces *atlas from the 'Atlas Build' list, then in the text box 'Does Mapping Span Exons Constraint' (which is to indicate that the peptide sequence is aligned to the exons on both sides of an intron) enter 'y', and select 'QUERY'. Rows of data are returned for the latest yeast build. That list can be saved and compared against a list of SGD introns to find that the atlas contains 13% of the 367 SGD yeast ORF introns. Two of these intron validations are for uncharacterized (experimentally unobserved) ORFs, YPR063C and YBL059C-A. To visualize the resulting information in the Ensembl Genome browser, the user may click on the coordinates in the results table (instructions on enabling the visualization are present on the navigation bar of the PeptideAtlas web pages).

Reverse look-ups of PeptideAtlas peptides are also possible from the Ensembl Genome browser website. For example, while browsing the genome view centered around the uncharacterized ORF YNL010W at [37] one can select the *S. cerevisiae *PeptideAtlas as a distributed annotation system (DAS) source, resulting in a large number of PeptideAtlas entries confirming portions of this predicted ORF sequence. Selecting any of these links can bring the user back to the PeptideAtlas summary pages for more detail.

## Conclusion

We have demonstrated that MS/MS spectra from a large number of diverse sources can be uniformly processed and combined to create a large dataset useful for exploring and validating the yeast proteome. The public interfaces allow creation of high quality peptide lists that can be used to synthesize reference molecules for targeted proteomics. The datasets are also useful to examine MS based proteomics in general, can be used for software development, and can be used by other researchers to validate hypothetical proteins for examples. The *S. cerevisiae *PeptideAtlas possesses the highest degree of proteome coverage for any eukaryotic organism to date in a single public database offering entire datasets as validation, and, as such, is a growing resource that will continue to improve as more researchers contribute data.

## Materials and methods

### Experiment processing

Some of the process level details of the experiment processing are provided here. The mzXML [20] conversions from vendor format files to mzXML files were performed using software available at our Sashimi software site [38]. The SEQUEST parameters used in MS/MS assignment were for semi-tryptic digestion and one static modification of methionine due to oxidation. Additional parameters were required for some datasets, such as those labeled with ICAT or samples treated with iodoacetemide. All sequest.params files can be found in the searched archive files at our repository [39] for the public datasets. (Datasets that researchers have requested to be kept as private until they have published are not present in the data repository, but are included in the PeptideAtlas database with minimum sample annotation.)

The PeptideProphet software is available at our Sashimi software site [23]. The SBEAMS database application and the Proteomics module and PeptideAtlas modules within it are available at our SBEAMS software site [40] as downloads from a subversion code repository. The code is browsable from the worldwide web [41]. The BLAST algorithm was used with the parameters for 100% identity matches to a small peptide in a protein reference database:

blastp -F F -W 2 -M PAM30 -G 9 -E 1 -e 10 -K 50 -b 50

## Additional data files

The following additional data are available with the online version of this paper. Additional data file 1 is a comma-separated text file containing a table of *S. cerevisiae *ORF fasta files used to create the reference database. The table includes the ftp addresses of the datasets and the number of ORFs unique to each dataset. Additional data file 2 is a comma-separated text file containing a table of the uncharacterized ORFs seen in *S. cerevisiae *PeptideAtlas. Included are the number of times their peptides were observed and the best PeptideProphet probability associated with their peptides. Additional data file 3 is a comma-separated text file containing a table of the dubious ORFs seen in *S. cerevisiae *PeptideAtlas. Included are the number of times their peptides were observed, and the best PeptideProphet probability associated with their peptides. Additional data file 4 is a comma-separated text file containing a table of the ORFs from transposable element genes seen in *S. cerevisiae *PeptideAtlas, the number of times their peptides were observed, and the best PeptideProphet probability associated with their peptides.

## Supplementary Material

Additional data file 1 A comma-separated text file. The table includes the ftp addresses of the datasets and the number of ORFs unique to each dataset. Click here for file

Additional data file 2 A comma-separated text file. Included are the number of times their peptides were observed and the best PeptideProphet probability associated with their peptides. Click here for file

Additional data file 3 A comma-separated text file. Included are the number of times their peptides were observed, and the best PeptideProphet probability associated with their peptides. Click here for file

Additional data file 4 A comma-separated text file. Included are the number of times their peptides were observed, and the best PeptideProphet probability associated with their peptides. Click here for file

## Figures and Tables

**Table 1:** List of experiments

Experiment	Instrument	Treatment/labeling	Separation	Strain	Data contributors	Affiliation	Reference
gricat	LCQ Classic	ICAT	SCX	BY4741	J Ranish, T Ideker	ISB	[42]
cdc15_cdc23_newICAT	LCQ DECA	clICAT	SCX	BY4742	B Raught	ISB	-
cdc15_cdc23_oldICAT	LCQ DECA	ICAT	SCX	BY4742	B Raught	ISB	-
cdc15_cdc23_ICAT	LCQ DECA	clICAT	SCX	BY4742	B Raught	ISB	-
cdc23_amf_newICAT	LCQ DECA	clICAT	SCX	BY4742	B Raught	ISB	-
contFFE2Murea	LCQ DECA	clICAT	SCX	BY4741 ?	F Kregenow, R Aebersold	ISB	-
FFEY1	LCQ DECA	FFE	Unknown	BY4741 ?	F Kregenow, R Aebersold	ISB	-
FFEY1Scx	LCQ DECA	FFE	SCX	BY4741 ?	F Kregenow, R Aebersold	ISB	-
FFEY2	LCQ DECA XP	FFE	Unknown	BY4741 ?	F Kregenow, R Aebersold	ISB	-
PeteryeastIcatstdFFE	LCQ DECA XP	clICAT	Unknown	BY4741 ?	F Kregenow, R Aebersold	ISB	-
TSAAT000c	LCQ DECA XP	clICAT	SCX	BY2125	M Flory *et al*.	ISB	[43]
TSAAT030c	LCQ DECA XP	clICAT	SCX	BY2125	M Flory *et al*.	ISB	[43]
TSAAT060c	LCQ DECA XP	clICAT	SCX	BY2125	M Flory *et al*.	ISB	[43]
TSAAT090c	LCQ DECA XP	clICAT	SCX	BY2125	M Flory *et al*.	ISB	[43]
TSAAT120c	LCQ DECA XP	clICAT	SCX	BY2125	M Flory *et al*.	ISB	[43]
TSAAxT000old	LCQ DECA	ICAT	SCX	BY2125	M Flory	ISB	[43]
T00	LCQ DECA XP	ICAT	SCX	BY2125	M Flory	ISB	[43]
T30	LCQ DECA XP	ICAT	SCX	BY2125	M Flory	ISB	[43]
T50	LCQ DECA XP	ICAT	SCX	BY2125	M Flory	ISB	[43]
opd00034_YEAST	LCQ DECA XP	None	SCX	DBY8724	P Lu	University of Texas	[44]
opd00035_YEAST	LCQ DECA XP	None	SCX	DBY8724	P Lu	University of Texas	[44]
peroximalPrep0702	LCQ Classic	ICAT	SCX	BY4743	M Marelli *et al*.	ISB	[45]
Comp12vs12sizefrac	LCQ DECA	Iodoacetemide	SCX	BY4741	DB Martin	ISB	-
pxproteome	LCQ DECA	clICAT	SCX	BY4743	M Marelli *et al*.	ISB	[45]
Comp12vs12standSCX	LCQ DECA	Iodoacetemide	SCX	BY4741	DB Martin	ISB	-
YeastICAT	LCQ Classic	ICAT	SCX	Derivative of BY4741	J Ranish	ISB	-
PvM1	LCQ Classic	ICAT	SCX	BY4743	M Marelli *et al*.	ISB	[45]
peroximal_clICAT	LCQ Classic	clICAT	SCX	BY4743	M Marelli *et al*.	ISB	[45]
Ac30	LCQ DECA XP	clICAT	SCX	BY1782, BY2125	KR Serikawa *et al*.	University of Washington	[46]
yeast	LCQ DECA	Iodoacetemide	SCX	YPH499	Gygi *et al*.	Harvard Medical School	[47]
gel_msms	LCQ DECA	Iodoacetemide	Gel, SCX	BY4742	Ho *et al*.	MDS Proteomics	[30]
mudpit	LCQ DECA XP PLUS	Iodoacetemide	SDS-PAGE, SCX	YRP480	P Haynes *et al*.	University of Arizona	[48]
ipg_ief	LCQ DECA XP PLUS	Iodoacetemide	SDS-PAGE, IEF	YRP480	P Haynes *et al*.	University of Arizona	[48]
rp_int_selected	LCQ DECA XP PLUS	Iodoacetemide	SDS-PAGE	YRP480	P Haynes *et al*.	University of Arizona	[48]
rp_mass_selected	LCQ DECA XP PLUS	Iodoacetemide	SDS-PAGE	YRP480	P Haynes *et al*.	University of Arizona	[48]
sdspage	LCQ DECA XP PLUS	Iodoacetemide	SDS-PAGE	YRP480	P Haynes *et al*.	University of Arizona	[48]
YM_N14N15_DAYGly	LCQ Classic	None	Unknown	DBY8724	P Lu	University of Texas	[44]
YM_N14N15_DAYSer	LCQ Classic	None	Unknown	DBY8724	P Lu	University of Texas	[44]
YM_N14N15_SCYGly	LCQ Classic	None	Unknown	DBY8724	P Lu	University of Texas	[44]
YM_N14N15_SCYSer	LCQ Classic	None	Unknown	DBY8724	P Lu	University of Texas	[44]
APEX_04-22	LCQ Classic	None	Unknown	DBY8724	P Lu	University of Texas	[44]
APEX_04-23	LCQ Classic	None	Unknown	DBY8724	P Lu	University of Texas	[44]
APEX_04-24	LCQ Classic	None	Unknown	DBY8724	P Lu	University of Texas	[44]
APEX_04-28	LCQ Classic	None	Unknown	DBY8724	P Lu	University of Texas	[44]
YeastSCXReps	LCQ Classic	Iodacetimide	SCX	BY4741	Maynard *et al*.	NIH	[49]
FFE_nonICAT	LCQ Classic	Iodacetimide	FFE	BY2125?	Mingliang Ye	ISB	

clICAT is acid cleavable ICAT D0227/D9236 and ICAT is the older ICAT reagent D0422/D8450. FFE, free flow electrophoresis; IEF, is isoelectric focusing; SCX, strong cation exchange; SDS-PAGE, sodium dodecyl (lauryl) sulfate-polyacrylamide gel electrophoresis.

**Table 2:** The number of spectra acquired and peptides identified per experiment

Experiment	numSpec(P > 0.0)	numSpec(P > 0.9)	numDistinctPeptides(cumulative, P > 0.9)
gricat	24,833	2,585	734
cdc15_cdc23_newICAT	172,938	22,269	2,889
cdc15_cdc23_oldICAT	156,353	8,281	3,184
cdc15_cdc23_ICAT	21,392	3,337	3,293
cdc23_amf_newICAT	127,673	25,861	4,096
contFFE2Murea	105,686	5,473	4,430
FFEY1	64,880	2,966	5,994
FFEY1Scx	7,045	796	6,396
FFEY2	60,140	7,159	8,877
PeteryeastIcatstdFFE	136,563	4,680	9,375
TSAAT000c	217,919	43,628	11,423
TSAAT030c	249,774	42,519	12,042
TSAAT060c	214,851	28,089	12,424
TSAAT090c	198,057	31,428	13,247
TSAAT120c	179,408	26,182	13,588
TSAAxT000old	101,166	6,311	13,728
T00	94,208	9,206	13,840
T30	83,758	8,758	13,935
T50	106,572	4,606	14,026
opd00034_YEAST	24,049	4,554	14,557
opd00035_YEAST	23,715	3,931	14,862
peroximalPrep0702	24,209	3,197	15,236
Comp12vs12sizefrac	28,926	11,703	17,553
pxproteome	23,720	1,552	17,724
Comp12vs12standSCX	31,652	12,103	18,966
YeastICAT	55,922	6,760	19,577
PvM1	3,796	323	19,623
peroximal_clICAT	92,721	3,693	19,778
Ac30	255,937	37,873	20,025
yeast	140,567	29,411	24,548
gel_msms	343,654	43,022	32,450
mudpit	36,599	10,811	32,859
ipg_ief	136,209	27,792	33,383
rp_int_selected	53,358	14,669	33,684
rp_mass_selected	71,151	4,881	33,857
sdspage	92,802	19,208	34,734
YM_N14N15_DAYGly	38,951	1,658	34,806
YM_N14N15_DAYSer	39,315	1,862	34,843
YM_N14N15_SCYGly	37,124	1,364	34,861
YM_N14N15_SCYSer	37,658	2,238	34,902
APEX_04-22	38,334	1,732	34,926
APEX_04-23	38,175	1,775	34,949
APEX_04-24	36,407	1,359	34,963
APEX_04-28	40,612	2,248	34,995
YeastSCXReps	118,083	60,568	35,446
FFE_nonICAT	21,054	6,870	36,133

Column 1 is the experiment name, column 2 is the number of spectra associated with PeptideProphet probabilities >0, column 3 is the number of spectra associated with PeptideProphet probabilities >0.9, and column 4 is the cumulative number of distinct peptides with PeptideProphet probabilities >0.9.

**Table 3:** Statistics for the current *S. cerevisiae *PeptideAtlas

	P_limit _= 0.9, N_obs _> 0	P_limit _= 0.95, N_obs _> 0	P_limit _= 0.99, N_obs _> 0	P_limit _= 0.9, N_obs _> 1	P_limit _= 0.95, N_obs _> 1	P_limit _= 0.99, N_obs _> 1
# Experiments	47	47	47	47	47	47
# MS runs	2,579	2,579	2,579	2,579	2,579	2,579
# MS/MS	4.9 M	4.9 M	4.9 M	4.9 M	4.9 M	4.9 M
# MS/MS with P ≥ P_limit_	600,960	565,217	472,234	586,708	552,434	461,827
# Distinct peptides with P ≥ P_limit_	36,133	33,377	27,909	21,840	21,646	20,251
# Distinct peptides with perfect SGD alignment	35,434	32,790	27,499	21,469	21,281	19,942
# SGD ORFs seen in PeptideAtlas	4,249 (62%)	3,903 (57%)	3,476 (51%)	3,069 (45%)	3,049 (45%)	2,935 (43%)
# SGD ORFs unambiguously seen in PeptideAtlas	3,980 (59%)	3644 (54%)	3,224 (47%)	2,795 (41%)	2,778 (41%)	2,672 (39%)

The percentage of SGD ORFs seen in PeptideAtlas is shown as a function of lower limit PeptideProphet probabilities and number of times peptide has been observed above lower limit. Using the most generous parameters of the build, we see 62% of the SGD ORFs. As an aside, 68% of SGD ORFs have Systematic gene names and we observe 76% of those. This is comparable to the 83% of ORFs with Systematic gene names that Ghaemmaghami *et al*. [14] observed in their protein expression study.

**Table 4:** Expected errors

Case	Expected errors P_limit _= 0.9	Expected errors P_limit _= 0.95	Expected errors P_limit _= 0.99
MS/MS P_i _≥ P_limit_	0.00915 (9%)	0.00517 (5%)	0.00137 (1%)
MS/MS P_i _≥ P_limit_, N_peptide observed _> 1	0.00884 (9%)	0.00506 (5%)	0.00136 (1%)
Consensus peptide best P_i_, P_i _≥ P_limit_	0.01027 (10%)	0.00510 (5%)	0.00120 (1%)
Consensus peptide best P_i_, P_i _≥ P_limit_, N_peptide observed _>1	0.00272 (3%)	0.00215 (2%)	0.00078 (1%)

Expected errors in the *Saccharomyces *PeptideAtlas are calculated for four cases and lower probability limits (P_limit _= 0.9, 0.95, 0.99): the assigned probability P of each MS/MS is used for all P_i _≥ P_limit_; the assigned probability P of each MS/MS is used for all P_i _≥ P_limit _where the associated peptide has been seen in the *S. cerevisiae *PeptideAtlas more than once'; the best probability for each unique peptide sequence is used for all P_i _≥ P_limit_; the best probability for each unique peptide sequence is used for all P_i _≥ P_limit _when the peptide has been seen in the *S. cerevisiae *PeptideAtlas more than once.

**Table 5:** Numbers of proteins matching SGD ORF annotation categories

ORF annotation	SGD	PeptideAtlas P > 0.9, N_obs _> 0	PeptideAtlas P > 0.9, N_obs _> 1
Uncharacterized	1,414	695 (49%)	405 (29%)
Verified	4,366	3,250 (74%)	2,449 (56%)
Verified | silenced gene	4	1 (25%)	0 (0%)
Dubious	820	95 (12%)	13 (2%)
Pseudogene	21	4 (19%)	1 (5%)
Transposable element gene	89	18 (20%)	13 (15%)
Total	6,714	4,063 (61%)	2,881 (43%)

The percent of SGD ORFs seen in the PeptideAtlas are shown in columns 3 and 4. Column 3 uses all peptides with probabilities ≥ 0.9, while column 4 excludes peptides that have only been seen once in the S. cerevisiae PeptideAtlas.
